# Evolutionary Rescue of an Environmental *Pseudomonas otitidis* in Response to Anthropogenic Perturbation

**DOI:** 10.3389/fmicb.2020.563885

**Published:** 2021-01-18

**Authors:** Manuel II García-Ulloa, Ana Elena Escalante, Alejandra Moreno-Letelier, Luis E. Eguiarte, Valeria Souza

**Affiliations:** ^1^Departamento de Ecología Evolutiva, Instituto de Ecología, Universidad Nacional Autónoma de México (UNAM), Ciudad de México, Mexico; ^2^Laboratorio Nacional de Ciencias de la Sostenibilidad, Instituto de Ecología, Universidad Nacional Autónoma de México (UNAM), Ciudad de México, Mexico; ^3^Jardín Botánico, Instituto de Biología, Universidad Nacional Autónoma de México (UNAM), Ciudad de México, Mexico

**Keywords:** coalescence, Cuatro Cienegas, genomics, horizontal gene transfer, microbial ecology, population genetics, recombination, selection

## Abstract

Anthropogenic perturbations introduce novel selective pressures to natural environments, impacting the genomic variability of organisms and thus altering the evolutionary trajectory of populations. Water overexploitation for agricultural purposes and defective policies in Cuatro Cienegas, Coahuila, Mexico, have strongly impacted its water reservoir, pushing entire hydrological systems to the brink of extinction along with their native populations. Here, we studied the effects of continuous water overexploitation on an environmental aquatic lineage of *Pseudomonas otitidis* over a 13-year period which encompasses three desiccation events. By comparing the genomes of a population sample from 2003 (original state) and 2015 (perturbed state), we analyzed the demographic history and evolutionary response to perturbation of this lineage. Through coalescent simulations, we obtained a demographic model of contraction-expansion-contraction which points to the occurrence of an evolutionary rescue event. Loss of genomic and nucleotide variation alongside an increment in mean and variance of Tajima’s *D*, characteristic of sudden population expansions, support this observation. In addition, a significant increase in recombination rate (R/θ) was observed, pointing to horizontal gene transfer playing a role in population recovery. Furthermore, the gain of phosphorylation, DNA recombination, small-molecule metabolism and transport and loss of biosynthetic and regulatory genes suggest a functional shift in response to the environmental perturbation. Despite subsequent sampling events in the studied site, no pseudomonad was found until the lagoon completely dried in 2017. We speculate about the causes of *P. otitidis* final decline or possible extinction. Overall our results are evidence of adaptive responses at the genomic level of bacterial populations in a heavily exploited aquifer.

## Introduction

Anthropogenic perturbations alter evolutionary trajectories of bacterial populations by introducing novel and often strong selective pressures to biological systems (Davison, 1999; Krause et al., 2012; Tello et al., 2012; Evans and Wallenstein, 2014; Romero et al., 2019), in some cases leading to the breakdown of community dynamics and subsequent ecosystem collapse (Grant et al., 2017). The evolutionary response of species to these pressures depends on the standing genetic variation within populations, which allows for adaptation to the new environmental conditions (Holt, 1990).

Periodic selection is the main model that explains bacterial evolution (Cohan, 2001, 2005) and has been observed in multiple laboratory experiments on *Escherichia coli* (Atwood et al., 1951; Papadopoulos et al., 1999; Notley-McRobb and Ferenci, 2000), *Pseudomonas fluorescens* and *P. aeruginosa* in lung infections (Barrett et al., 2005; Caballero et al., 2015) and through time-series metagenomics in environmental aquatic *Chlorobium* and Bacteroidales (Bendall et al., 2016). Periodic selection consists on the succession or diversification of a bacterial population when a new advantageous variant appears, as it can either provide a fitness benefit within its current niche, out-competing its ancestors to extinction, thus sweeping through the population, or allow for the occupation of a new niche, generating a new ecotype (Cohan, 2001). This process applies well to mainly asexual bacterial populations in constant environments, where it has been mostly studied; evolution via periodic selection assumes that horizontal gene transfer (HGT) is extremely rare or completely absent (Cohan, 2005). However, this tends to not be the case in the face of a new environmental stress (Foster, 2005; Aminov, 2011).

In contrast, evolutionary rescue has been proposed as a process on which populations that suffer a sharp demographic decline as a consequence of environmental shock, escape from extinction by adaptive mechanisms (Orr and Unckless, 2008; Bell, 2013, 2017; Carlson et al., 2014). In evolutionary rescue, fast adaptive response can happen along with HGT, which accelerates genome evolution by the introduction of new advantageous genetic variation to populations (Jain et al., 2003; Pál et al., 2005; Baharoglu and Mazel, 2011; Baskett and Gomulkiewicz, 2011; Uecker and Hermisson, 2016). This creates a hallmark U-shaped demographic curve depicting the population decline and recovery, first predicted by Gomulkiewicz and Holt (1995) and later observed experimentally by others (Bell and Gonzalez, 2009; Agashe et al., 2011; Ramsayer et al., 2013).

Given the difficulties of *in situ* studies (Bell, 2017), evolutionary rescue in bacteria has only been studied *in silico* through mathematical models and simulations (Gomulkiewicz and Holt, 1995; Orr and Unckless, 2008, 2014; Uecker and Hermisson, 2016; Vogwill et al., 2016; Van Den Elzen et al., 2017; Anciaux et al., 2018, 2019; Carja and Plotkin, 2019) and in few laboratory experiments (Bell and Gonzalez, 2009; Agashe et al., 2011; Ramsayer et al., 2013). Long-term consequences of evolutionary rescue are is still under debate; for instance, does the resulting erosion in genetic diversity leaves populations vulnerable to extinction after evolutionary rescue (Carlson et al., 2014; Bell, 2017)?

Anthropogenic perturbation of natural environments are evolutionary experiments to look at the role of recombination and to evaluate periodic selection versus evolutionary rescue in bacterial populations. One worldwide example of an anthropogenic environmental perturbation is water overexploitation (Konikow and Kendy, 2005; Wada et al., 2010), which particularly affects arid ecosystems (Cui and Shao, 2005; McLendon et al., 2008). A dramatic example of this global trend is Cuatro Cienegas Basin (CCB), an oasis in the Chihuahua desert in northern Mexico. CCB is composed of various hydrological systems that used to include numerous pools, lagoons and rivers fed by underground springs (Minckley and Cole, 1968). Nowadays, there is only 5% or less of the original wetland left, as the overexploitation of groundwater through canals that have drained the wetlands since the 70’s has had a strong impact on CCB hydrological systems. Paradigm of this tragedy is the desiccation of the larger lagoon of our long-term study site, Churince (Souza et al., 2007, 2018; De Anda et al., 2018). This is particularly poignant, since Churince was described as a “lost world” (Souza et al., 2018), where unusually unbalanced stoichiometry (PO_4_ as low as 0.1 μM and often below detection and N:P > 200:1 for total nutrients) (Elser et al., 2005, 2018; Escalante et al., 2008; Lee et al., 2015, 2017; Ponce-Soto et al., 2015) had isolated this wetland from modern microbial communities, preserving a hyper diverse indigenous microbiota (Desnues et al., 2008; Moreno-Letelier et al., 2012; Gómez-Lunar et al., 2016; Souza et al., 2018; Taboada et al., 2018) composed of unique bacterial lineages of which a large fraction descended from marine ancestors (Souza et al., 2006; Alcaraz et al., 2008).

Previous studies of microbial populations of the Churince system have shown that the genus *Pseudomonas* have been impacted by past and recent environmental shocks of the Cuatro Cienegas aquatic system (Avitia et al., 2014; Ponce-Soto et al., 2015; García-Ulloa et al., 2019). Biology and ecology of pseudomonads from Churince has been described in different studies (Rodríguez-Verdugo et al., 2012; Avitia et al., 2014; Ponce-Soto et al., 2015) and a new lineage, *P. cuatrocienegasensis*, was previously described (Escalante et al., 2009). More recently two new strains of the *P. aeruginosa* PA14 clade native to Laguna Intermedia were sampled for the first time during the first desiccation event in 2011 (García-Ulloa et al., 2019). *Pseudomonas otitidis* isolated from CCB also represented a separate native lineage that was present, but rare, at Laguna Intermedia (Rodríguez-Verdugo et al., 2012). The availability of *P. otitidis* isolates from 2003 (normal state) and its subsequent isolation in 2015 (perturbed state) made it possible to observe the genomic evolution of this lineage in response to the desiccation events.

In this study we took advantage of a unique opportunity to study the evolution of natural populations of bacteria in the face of environmental perturbation. We studied the response of *P. otitidis* from Laguna Intermedia to a new selective pressure in the form of desiccation events. We used comparative genomics and population genetics techniques that allowed us to reconstruct the evolutionary response and demographic history of *P. otitidis* at Laguna Intermedia in a period of 12 years, from the first sampling (2003) to the last time it was found (2015), encompassing 3 desiccation events in 2011, 2012 and during the last sampling in 2015.

## Materials and Methods

### Study Site

Laguna Intermedia from the Churince system in Cuatro Ciénegas, Coahuila, Mexico (26.848572 N, 102.141783 W) suffered four recent desiccation events: October 2011, November 2012, October 2015 and October 2016 before completely drying up in 2017 (Souza et al., 2018; García-Ulloa et al., 2019). Churince was a long-term study site, and water physico-chemical conditions were recorded periodically since 2002 with the multiparameter water quality sonde Hydrolab MS5. In Laguna Intermedia, salinity, specific conductivity and pH were particularly altered with each desiccation event (Supplementary Figure 1).

### Sampling and Isolate Collection

On October 2015, surface water samples were taken from multiple points of Laguna Intermedia using sterile BD Falcon vials (BD Biosciences, San Jose, CA, United States). Isolates were obtained by plating 200 μl of each sample on *Pseudomonas* Isolation Agar (PIA) plates, which were further incubated at 32°C for 24 h. Individual colonies were transferred to new PIA plates and grown at 32°C for 1 day. Each colony was grown in LB medium at 32°C for 1 day and then stored in 50% glycerol solution at −80°C. From October 2015 until 2017, when Laguna Intermedia became completely dry, no other pseudomonad was found, even if they were intensively looked after in different subsequent samplings and cultures through the implementation of this exact same protocol and sampling effort.

### Isolate Identification

DNA extraction was done with Qiagen DNeasy Blood & Tissue kit. 16S rRNA gene was amplified with universal primers 27F and 1492R and sequenced by the Sanger method. 138 colonies from the October 2015 sampling were screened and 7 of them were identified as *P. otitidis* through a blast search against the RefSeq database. Additionally, all 5 existing isolates obtained from water in August 2003 previously identified as *P. otitidis* by Avitia et al. (2014) were used for the analysis as representatives of the undisturbed state of the lagoon.

### Sequencing, Assembly and Genome Annotation

Sequencing was carried out using the Illumina MiSeq platform in its PE 2x300 modality, obtaining a coverage larger than 30× for all genomes (Supplementary Table 1). Genomes were assembled de novo with MaSuRCA 3.2.4 using default settings (Zimin et al., 2013), followed by SPAdes 3.10.0 using the prior assembly with the *–trusted_contigs* option for refinement (Bankevich et al., 2012). Contigs less than 600 bp long and 10× of k-meric coverage were discarded. The quality and integrity evaluation of the assemblies was done with Quast v4 (Gurevich et al., 2013) and BUSCO v2 (Simão et al., 2015; Supplementary Table 1). All genomes were deposited on NCBI with the accession numbers JAANPJ000000000 (41M), JAANPK000000000 (39M), JAANPL000000000 (38M), JAANPM000000000 (36M), JAANPN000000000 (35M), JAANPO000000000 (34M), JAANPP000000000 (32M), JAANPQ000000000 (15M), JAANR000000000 (13M), JAANPS000000000 (12M), JAANPT000000000 (10M), JAANPU000000000 (9M). A database with all the complete annotated genomes of *Pseudomonas* to date was built as reference for annotation with Prokka 1.11 (Seemann, 2014).

### Genealogy of Core Genomes

Phylogenomic relationships between all lineages were assessed through a core genome genealogy. A genome alignment was done with progressiveMauve using default settings and the core genome was extracted with the stripSubsetsLCBs script (Darling et al., 2010). Core genome genealogy was performed with FastTree2.1.10 (Price et al., 2010) using the gamma and general time-reversible model parameters. NCBI accession numbers for the reference genomes used in the genealogy are: AE008922.1 (*Xanthomonas campestris*), NC_012660.1 (*P. fluorescens* SBW25), NC_002947.4 (*P. putida* KT2240), NZ_PXJI00000000.1 (*P. otitidis* PAM-1), NC_002516.2 (*P. aeruginosa* PAO1) and NZ_FOFP00000000.1 (*P. cuatrocienegasensis*).

### Demographic Analysis

The raw genomic reads were filtered by a quality of 20 with Sickle v1.33 (Joshi and Fass, 2011) and mapped against the genome of *P. otitidis* PAM-1 with the Burrows-Wheeler aligner (Li and Durbin, 2009). SNP calling was done using Genome Analysis Toolkit (Van der Auwera et al., 2013), SAMtools (Li et al., 2009) and VarScan2.3.9 (Koboldt et al., 2012) programs. The folded frequency spectrum was calculated with easySFS^1^.

Coalescence simulations were performed with fastsimcoal2 (Excoffier et al., 2013); 500 repetitions of 500,000 simulations were carried out with 40 optimization cycles and 1,000 parametric bootstraps of the best model to obtain 95% confidence intervals. Demographic models allowed the possibility of positive (expansion: E) and negative (contraction: K) population changes as well as absence of changes (constant population size: C) in three time intervals based on the desiccation events (August 2003 to October 2011; October 2011 to November 2012; November 2012 to October 2015) based on the dates of the desiccation events, resulting in 27 different models. The initial parameters used in the models were: generational time of *P. otitidis* sister clade, *P. aeruginosa*, in low phosphorus medium = 0.04 h-1 (Buch et al., 2008), range of generations from 2003 to 2015 = 6,300 to 2,100 (this study), mutation rate of non-mutator *P. aeruginosa* = 2.5 e-8 (Oliver et al., 2000), recombination rate calculated from R/θ = 5 e-9 (this study, using the mentioned mutation rate), effective population size range = 500,000 to 8,000,000 calculated using a coalescent-based approach on 4 concatenated housekeeping genes (*acnB*, *gyrB*, *recA* and *rpoD*) (Avitia et al., 2014). The evaluation of the models was done by calculating and comparing their weighted AIC values (wAIC) which serve as a measure of the strength of each model in comparison to the others given their likelihoods, from 0 to 100%.

### Pan-Genomic and Functional Analysis

Global core, cores by sampling, exclusive accessory and shared accessory genomes were obtained with the consensus of the three clustering algorithms (BDBH, OMCL and COG) of GET_HOMOLOGUES (Contreras-Moreira and Vinuesa, 2013) and aligned with ClustalW using default settings (Thompson et al., 1994). The orthologous genes of the global core genome were used to obtain the mean nucleotide diversity obtained from all possible pairwise comparisons within each cluster of orthologous group (π) and mean Tajima’s *D* with DnaSP v5 (Librado and Rozas, 2009). Tajima’s *D* is often used in population genetics studies to assess selection and demographic changes in populations comparing the pairwise differences with the number of segregating sites. From a demographic perspective, a neutral value of Tajima’s *D* signals constant population size, while a positive and negative values indicate a recent population contraction or expansion, respectively.

Functional annotation of the genes of the exclusive accessory genomes was done with Blast2GO (Götz et al., 2008), using Biological Process gene ontology category for the functional analysis. Furthermore, a protein blast against the non-redundant protein sequences database was performed on the translated genes from the exclusive accessory genomes.

Rarefaction curves of the unique gene clusters were done with the R-package micropan v 2.0.1 (Snipen and Liland, 2015) using its dclust algorithm on hmmer domains of the gene products of the accessory genomes of each sampling separately. 1,000 permutations were performed.

### Recombination Estimation

To assess the changes in the importance of HGT, recombination rate to mutation ratio (R/θ), insert size (δ), number of substitutions introduced per insert (δν) and the effect of recombination to mutation on genome level variation (*r/m*) were calculated with ClonalFrameML using default settings (Didelot and Wilson, 2015). 1,000 pseudoreplicates were performed to obtain 95% confidence intervals.

## Results

### Evolutionary Rescue of *P. otitidis*: An Ephemeral Escape From Extinction

**FIGURE 1:**
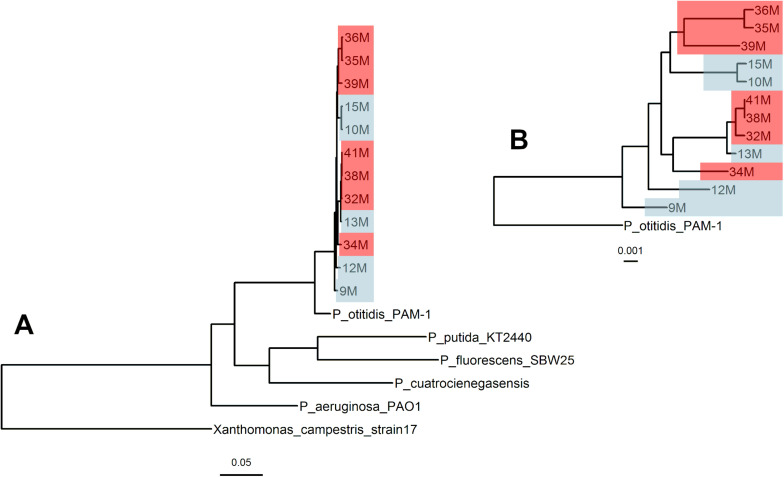
Genealogy of core genomes of *Pseudomonas otitidis* collected from Laguna Intermedia in the Churince system. Genealogy of the *Pseudomonas* genus including multiple outgroups **(A)** and zoomed at the clade that comprises the Churince isolates from this study **(B)**. The isolates from August 2003 (original state) are in blue and from October 2015 (perturbed state) in red. The bar below the trees represents branch lengths. Both phylogenies were calculated by FastTree2.0. All genomes were aligned with progressiveMauve and the core genome was extracted with the stripSubsetsLCBS script (Darling et al., 2010).

The genealogy of core genomes shows phylogenetic correspondence between isolates from 2003 and 2015, confirming their close relatedness which makes comparison between them valid (Figure 1). The lack of divergence regardless of the sampling date, as seen on the lack of separation within their phylogenetic group, points to all isolates forming part of the same population at different time points. In addition, the genealogy confirms the proximity of all isolates with *P. otitidis* PAM-1.

**FIGURE 2:**
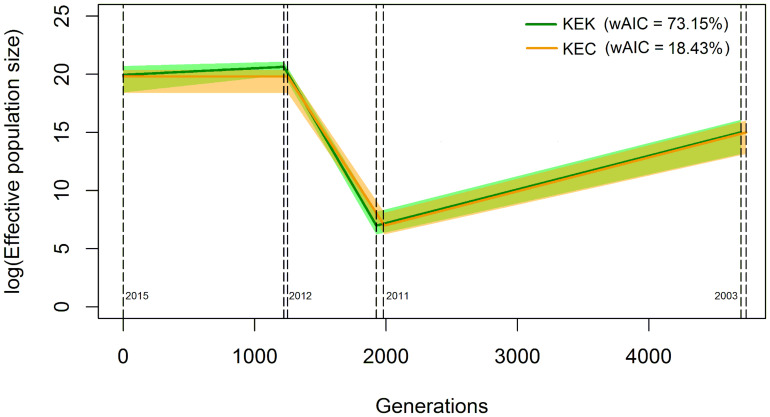
Graphical representation of the best two demographic models according to their weighted AIC value based on the results obtained from fastsimcoal2. Coalescent simulations were performed using the observed allele frequency spectrum of the *Pseudomonas otitidis* population from 2015, taking into account an initial population range of 500,000 to 8,000,000 estimated by Avitia et al. (2014), mutation rate of *P. otitidis* sister clade *P. aeruginosa* (wild tyfpe) of 5e-9 calculated by Oliver et al. (2000), generational time of *P. aeruginosa* in low phosphorus medium of 0.04 h-1 calculated by Buch et al. (2008), and range of generations from 2003 to 2015 of 6,300 to 2,100 and recombination rate R = 2.5 e-8 calculated from R/θ in this study. The solid colored lines are the average values and the shaded areas represent the 95% confidence intervals. Contraction-Expansion-Contraction (KEK) is the best model with a wAIC value of 73.15% (green) and Contraction-Expansion-Constant (KEC) follows with 18.45% (orange). Dotted vertical lines mark the desiccation events according to the generations calculated by fastsimcoal2. The population contraction from 2003 to 2011 for both models was approximately 99%. For detailed values see Supplementary Table 2.

The two best coalescent models according to their wAIC values are contraction-expansion-contraction (KEK, wAIC = 73.15%) and contraction-expansion-constant (KEC, wAIC = 18.43%), both coinciding in an initial population contraction from 2003 to the first desiccation event in 2011 of more than 99%, followed by a drastic population increase until the second desiccation event in 2012 (Figure 2 and Supplementary Table 2). Both models also reach a similar final population size in 2015, which is approximately 1,000 times larger than its initial size (Supplementary Table 2).

**FIGURE 3:**
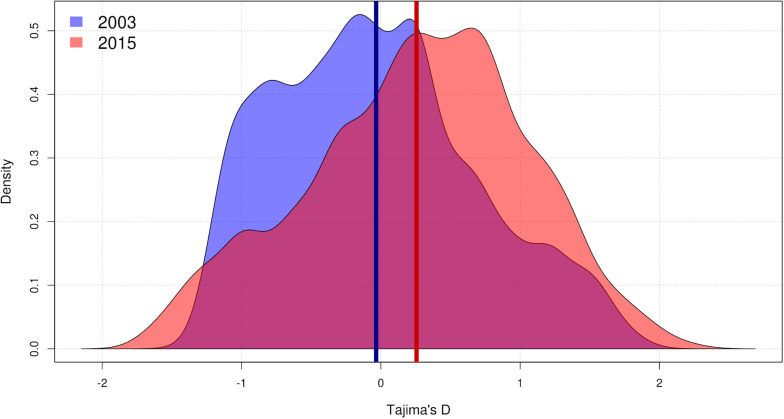
Density plot of the distribution of *Pseudomonas otitidis* Tajima’s *D* at core genome level. Samples of *P. otitidis* from 2003 and 2015 consist of 5 and 7 individuals, respectively. 3503 genes were used for the analysis due to the rest of the 3682 global core lacking any variation to measure. Kernel smoothing was used to plot the values. Vertical bars represent the means: –0.03 in 2003 (blue, left-skewed) and 0.26 in 2015 (red, right-skewed). The increase in mean value and the increase in variance are significant (paired two-sided Wilcoxon test: *V* = 2.07e6, *p* < 0.001; Lavene’s test: *F*(1,7004) = 23.8, *p* < 0.001).

There was a significant increase in mean Tajima’s *D*, which went from a marginally negative, almost neutral value (*D* = −0.03) in 2003 to a positive value (*D* = 0.26) in 2015 (paired two-sided Wilcoxon test: *V* = 2.07e6, *p* < 0.001). Variance in Tajima’s *D* increased from 0.50 to 0.61, and the change was also significant (Lavene’s test: *F*(1,7004) = 23.8, *p* < 0.001) (Figure 3).

### Genomic and Nucleotide Diversity Decreased After the Perturbation

**FIGURE 4:**
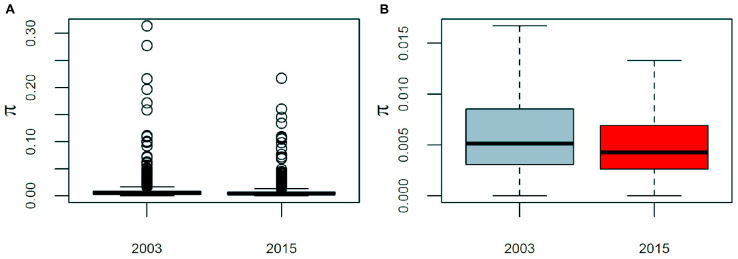
Boxplots of the average nucleotide diversity (π) of *Pseudmonas otitidis* at the global core genome level. The global core genome consists of 3,682 genes. Barplots are presented with **(A)** and with outliers (values outside 1.5 times the interquartile range) **(B)**. The change of mean and variance of π are significant (paired two-sided Wilcoxon test: *V* = 4.42e6, *p* < 0.001; Brown-Forsythe test: *F*(1, 7362) = 27.6, *p* < 0.001).

*Pseudomonas otitidis* isolated from Laguna Intermedia suffered a significant decrease both in its genomic and mean nucleotide diversity (π). The core genome increased by 17.3% (559 genes), while the accessory decreased by 32.8% (486 genes). Similarly, mean π of the global core genome (3,682 genes) decreased from 0.0075 to 0.0058, which represents a significant loss of 22% of its mean π (paired two-sided Wilcoxon test: *V* = 4.42e6, *p* < 0.001), as well as a significant decrease in its variance from 1.39e-4 to 6.4e-5 (Brown-Forsythe test: *F*(1, 7362) = 27.6, *p* < 0.001) (Figure 4).

### The *P. otitidis* Functional Shift in a Perturbed Environment

**FIGURE 5:**
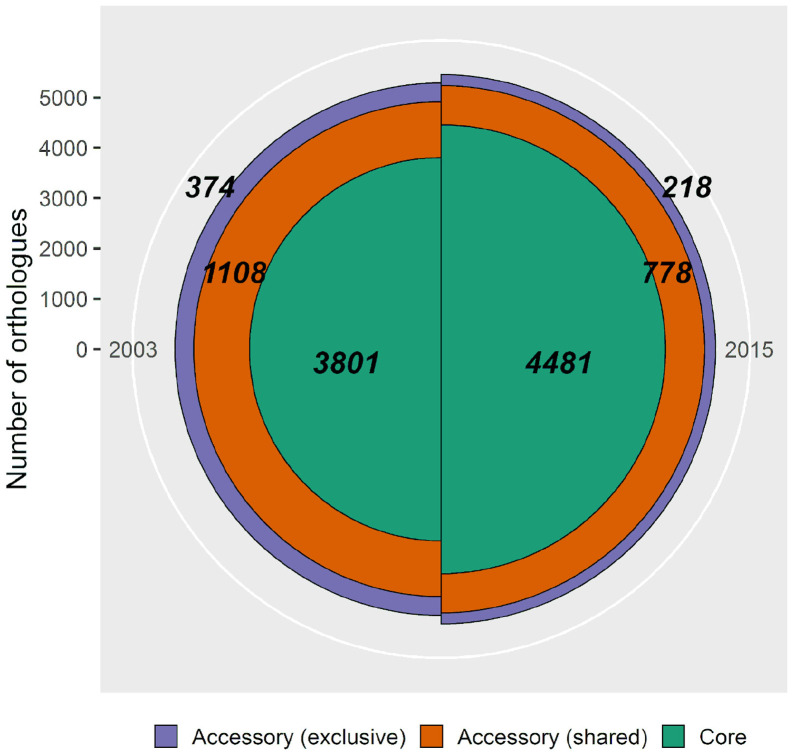
Pan-genomic comparison of *Pseudomonas otitidis* from 2003 and 2015 from Laguna Intermedia in the Churince system. The population from 2003 consists of 5 individuals and has a pan-genome of 5,283 genes and the one from 2015 consists of 7 individuals with a pan-genome of 5,477 genes. The green (innermost) fraction represents the core genome, the orange (middle) one the shared accessory genome and the purple one (outermost) the exclusive accessory genome of each sampling. Bold numbers indicate the quantity of genes in each category.

The exclusive accessory genome comprises the unique genes of each sampling, those that were lost (only found in 2003) and gained (only found in 2015), with a net decrease of 41.7% (156 genes). Exclusive accessory genomes of both samplings (Figure 5, purple fraction) were functionally annotated (Supplementary Tables 3, 4), with 27.8% of genes (104 genes) for the population of 2003, and 36.7% (80 genes) for the population of 2015 being functionally identifiable under the Biological Process gene ontology category.

**FIGURE 6:**
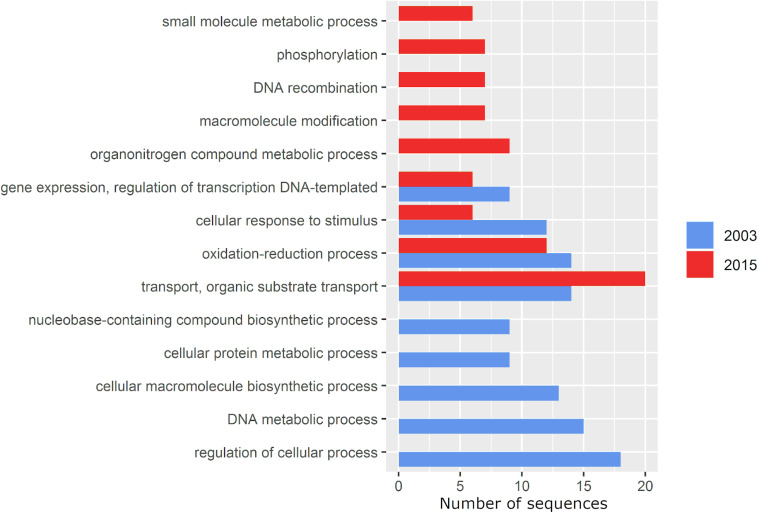
Multi-level gene ontology graph of the exclusive accessory genome of *P. otitidis* under the Biological Process category calculated by Blast2GO. The gene ontology terms (functions) are on the Y axis and the X axis represents the number of gene sequences assigned to each one. The 2003 and 2015 samplings are marked in blue and red, respectively. Bars alone represent functions lost (2003, blue) and gained (2015, red). Specific functions and annotation details are in Supplementary Tables 3, 4.

According to a multi-level functional analysis performed by Blast2GO, gained genes included functions related to metabolism of organic nitrogen compounds and small molecules, modification of macromolecules, phosphorylation and recombination. In contrast, genes involved in DNA and cellular metabolic process, biosynthesis of cellular macromolecules and nucleobase-containing compounds and regulation of cellular process were lost (Figure 6). Even though all other functional categories decreased in their number of genes, genes with transport functions, particularly of organic substrates, increased by 42.8% (Figure 6).

Blast searches showed an increase of accessory proteins of 3.4% from multiple closely related *P. otitidis* strains, in contrast with a decrease of 3.5% in proteins from other *Pseudomonas* species (2003: *P. aeruginosa*, *P. syringae*, *P. stutzeri*, *P. oleovorans*, *P. citronellolis*, *P. asplenii*, *P. alcaligenes* and *P. corrugata*; 2015: *P. aeruginosa*, *P. panipatensis*, *P. xanthomarina*, *P. alcaligenes*, *P. syringae*, *P. compostae* and *P. pseudoalcaligenes*). Proteins from other genera were less than 1% on either sampling, although with a slight increase of 0.1% in 2015. Best hits of proteins acquired from other genera point to them originally belonging to *Sphaerotilus natans* subsp. *natans* DSM 6575, *Burkholderia gladioli* and *B. pseudomallei* MSHR3709 in the 2003 sampling and *Magnetospirillum marisnigri* and *Acinetobacter junii* in 2015. Only the *A. junii* protein has a known function as a fatty acid desaturase.

### Recombination Increased in a Less Diverse *P. otitidis* Population

Significant decreases in average size of recombinant fragments and substitutions per fragment reflect the loss in genetic diversity as seen in the decrease of δ by 23.6% (110 pb, CI 95% = 0.085 to 84 pb, CI 95% = 0.063; *p* > 0.001) and δν by 19.2% (5.5, CI 95% = 0.0043 to 4.5, CI 95% = 0.0035; *p* > 0.001). Moreover, an increase in recombination rate is depicted by a slight but significant increase in R/θ (0.19, CI 95% = 0.00017 to 0.20, CI 95% = 0.00018; *p* > 0.001). As a result, *r/m* also decreased significantly by 12.4% (1.05, CI 95% = 0.0011 to 0.92, CI 95% = 0.0009; *p* > 0.001) as the population became less genetically diverse and recombination occurred more often between more genetically similar individuals, thus, minimizing the role of recombination on the introduction of novel genetic variation.

## Discussion

According to the two best demographic models calculated by fastsimcoal2 (Excoffier et al., 2013), a population bottleneck drove *P. otitidis* near to extinction in 2011, followed by a drastic population expansion that peaked in 2012 (Figure 2). Although they differ on their last time interval (2012 to 2015; contraction vs. constant) the final population size in 2015 was about 1000 times greater than its initial size in 2003 in both models, with the 95% CI overlapping at all times (Figure 2 and Supplementary Table 2). An increase in mean Tajima’s *D* and its variance were also observed, supporting the observation of a population (Maruyama and Fuerst, 1985; Schmidt and Pool, 2002).

Another observable effect of evolutionary rescue is the loss of variation, since often a very small portion of the population survives and expands (Carlson et al., 2014; Bell, 2017). Accordingly, pan-genome analysis showed a decrease in both genomic variation (Figure 5) and mean π and its variance (Figure 4).

Rarefaction curves of the pan-genome unique gene clusters (Supplementary Figure 2; Snipen and Liland, 2015; Adamek et al., 2018) show that the 2015 population sample nearly reached its asymptote, while the 2003 sample did not. This observation points to a larger accessory genome in 2003 than the one we found (Figure 5) and, although its size is unknown, it is safe to assume it was larger than the one of 2015 (Supplementary Figure 2). As the 2003 sample may be incomplete in terms of genetic variation, conclusions regarding this matter must be taken with caution, since there are actually more lost genes than the ones found and there is a possibility that some of those genes are the ones we catalog as “gained” in 2015 but were not found in 2003 because of the small sample size (*n* = 5). Nonetheless, this result supports the observed decrease in diversity discussed before and, even though the 2003 population sample included the whole exclusive accessory genome from the 2015 sample, that would mean the latter is a subset of the former, which is consistent with an evolutionary rescue event.

The role of adaptive gene loss in bacteria has been previously studied (Hottes et al., 2013). Here, genes involved in regulation of transcription and cellular processes, response to stimulus, oxidation-reduction, biosynthesis of macromolecules and nucleobase-containing compounds and metabolism of DNA and proteins were lost (Figure 6). Loss of biosynthetic genes in bacteria is a general and well documented phenomenon by which lineages become auxotrophic in new environmental conditions due to the cost of maintaining genes that are no longer needed, thus becoming unable to produce some of the metabolites they needed to grow in their former environment (D’Souza et al., 2014). This can also be an adaptive response when the corresponding metabolite is sufficiently present in the environment (Morris et al., 2012). We believe the disruption of the original community interactions and their microbial “market” (Werner et al., 2014), may have rendered biosynthesis of certain metabolites an unnecessary burden, thus being lost as an adaptive response (Giovannoni et al., 2005). Albalat and Cañestro (2016) pointed out the importance of loss of regulatory functions in the adaptation of bacterial populations to new environments that require alternative or new metabolic pathways, which could be the case at Laguna Intermedia, given the increasing severity of the perturbations.

Functions gained include DNA recombination and phosphorylation, both of which have been observed to play a role in environmental stress response (Stock et al., 1989; Sutton et al., 2000; Lipa and Janczarek, 2020). Although a correlation cannot be drawn from our results, the gain of genes involved in DNA recombination coincides with a slight but significant increase in recombination rate (R/θ, 0.19 ± 0.00017 to 0.20 ± 0.00018, *p* < 0.001), suggesting a mechanism of evolutionary rescue assisted by HGT. Bacteria such as *Escherichia coli* (Little and Mount, 1982), *Vibrio* (Baharoglu and Mazel, 2011), *Staphylococcus* (Anderson et al., 2006) and *Pseudomonas* (Penterman et al., 2014) also possess a stress response known as SOS that consists of repairing DNA damage caused by multiple sources of stress, such as UV light, fungal metabolites, reactive oxygen species (Sutton et al., 2000), exposure to antibiotics and phages (Torres-Barceló and Hochberg, 2016). The SOS response could facilitate evolutionary rescue by integrating exogenous DNA into the genome. Contrastingly, *r/m* decreased significantly (12.4% decrease, *p* < 0.001), suggesting that although recombination increased, it occurred between a less diverse gene pool. This observation coincides with the general loss of genetic diversity, the less taxonomically diverse sources of the exclusive accessory genome in 2015, and that closer bacterial lineages tend to recombine more frequently (Nandi et al., 2015; Vazquez-Rosas-Landa et al., 2020).

Phosphorylation is a key mechanism involved in signal transduction as a response to environmental stimuli such as nitrogen deprivation, phosphorous availability, osmolarity and chemotaxis molecules (Stock et al., 1989). Experimental, experimental studies in rhizobia indicate that phosphorylation plays an essential role in many physiological processes in adaptation to various environmental factors (e.g., dicarboxylate transport, phosphate utilization and adaptation to pH stress and microaerobic conditions) (Liu et al., 2015) through multiple regulatory mechanisms (Lipa and Janczarek; 2020). As Laguna Intermedia became perturbed, the gain of phosphorylation-related genes may have helped *P. otitidis* to withstand the new environmental conditions and adapt to them. Additionaly, the increase in small-molecule metabolism genes correlates with an increase in metabolic versatility required under conditions of high competition (Torsvik et al., 2002), and a large variety of nutrients (Cases et al., 2003). Although no measurements of nutrients were taken in 2015, studies have found that cycles of hydration-desiccation in soil provide extra nutrients to surviving bacterial populations through bacterial remnants and osmolites (Bottner, 1985; Kieft et al., 1987; Fierer and Schimel, 2003; Št′ovíček et al., 2017), which may have been the case in Laguna Intermedia. We think that this change in nutrients translated in the transport of organic substrates functions being the only shared category of the exclusive accessory genome between samplings that increased (42.8%) in 2015 as an adaptation to a less oligotrophic Laguna Intermedia.

### Beyond Evolutionary Rescue

Together with the severity of the perturbation and population size (Gomulkiewicz and Holt, 1995), initial genetic diversity plays an important role in the survival of a population through evolutionary rescue (Ramsayer et al., 2013). Culturable bacterial lineages from the Churince system have been found to be highly clonal (Cerritos et al., 2011) and *P. otitidis* from Laguna Intermedia is no exception, with consistently low values of π and R/θ. In fact, clinical *P. aeruginosa* is considered clonal even at R/θ = 0.54 (Castañeda-Montes et al., 2018), which is almost 3 times the R/θ of *P. otitidis* of Laguna Intermedia.

Although the initial variation was enough to withstand the perturbation and enter evolutionary rescue, *P. otitidis* was not found after the 2015 desiccation event, even though subsequent attempts with the same sampling effort and protocol were done in March and October (same time of the year as the 2015 sampling) 2016. Bell (2017) has speculated that the loss of genetic variation after an evolutionary rescue event could render populations less likely to survive a subsequent (new) stress in fluctuating environments, as Laguna Intermedia became since 2011. However, *P. otitidis* only went through one bottleneck in the first desiccation event in 2011, not in the one of 2012 nor 2015. We propose two possible (non-mutually exclusive) explanations:

(1) Although population size remained high at the 2nd and 3rd desiccation events, we don’t know if genomic and/or nucleotide variation was being lost. One possible scenario is that the variation of *P. otitidis* progressively decreased to the point of it being incapable of responding to any new environmental pressure, as Bell (2017) proposed, and after going through three consecutive desiccation events, the fourth one in 2016 (Supplementary Figure 1) was the final nail in the coffin.
(2) The volume of water of Laguna Intermedia decreased with each desiccation event, which is reflected in the progressive increase in salinity, specific conductivity and pH shown in Supplementary Figure 1. In this scenario, the successive desiccation events could have progressively degraded the lagoon to the point of it becoming uninhabitable by *P. otitidis*, or harsh enough (on its environmental conditions, community dynamics or both) to keep *P. otitidis* at such low abundance that it could not be sampled again. Interestingly, in October 2016, using the same *Pseudomonas* isolation agar and the same sampling protocol with the same sampling effort, we only recovered multiple strains of environmental *Vibrio cholerae* for the first time ever in Laguna Intermedia. This serves as evidence that there was at least an important change in the community, supporting this scenario. Also, additional evidence of community disruption is provided by the finding of taxonomically very different non-pseudomonad genes on the exclusive accessory genomes, with 2 genes from *Burkholderia* and 1 from *Sphaerotilus* in 2003, and 1 from *Magnetospirillum* and 1 from *Acinetobacter* in 2015. Medium to long-term evolutionary rescue studies comparing constant and fluctuating environments with more samplings in time are needed in order to answer this question.

## Data Availability Statement

The datasets presented in this study can be found in online repositories. The names of the repository/repositories and accession number(s) can be found in the article/Supplementary Material.

## Author Contributions

MG-U, AE, AM-L, LE, and VS conceived and designed the study. MG-U and AE performed the fieldwork. MG-U performed the experimental procedures, analyzed all the data and wrote the original draft of the manuscript. VS and LE contributed with reagents, materials, computational resources, and acquired the funding for the project. AE, AM-L, LE, and VS supervised the experimental work and reviewed and edited the manuscript. All authors approved the final manuscript.

## Conflict of Interest

The authors declare that the research was conducted in the absence of any commercial or financial relationships that could be construed as a potential conflict of interest.

## Footnotes

1 https://github.com/isaacovercast/easySFS
