# Supplementary material for: Evolutionary Rescue of an Environmental Pseudomonas otitidis in Response to Anthropogenic Perturbation
Source: Front Microbiol. 2021 Jan 18;11:563885. doi: 10.3389/fmicb.2020.563885 (PMC7856823; doi:10.3389/fmicb.2020.563885)
Supplement: Supplementary file 1 [file Image_1.PDF]

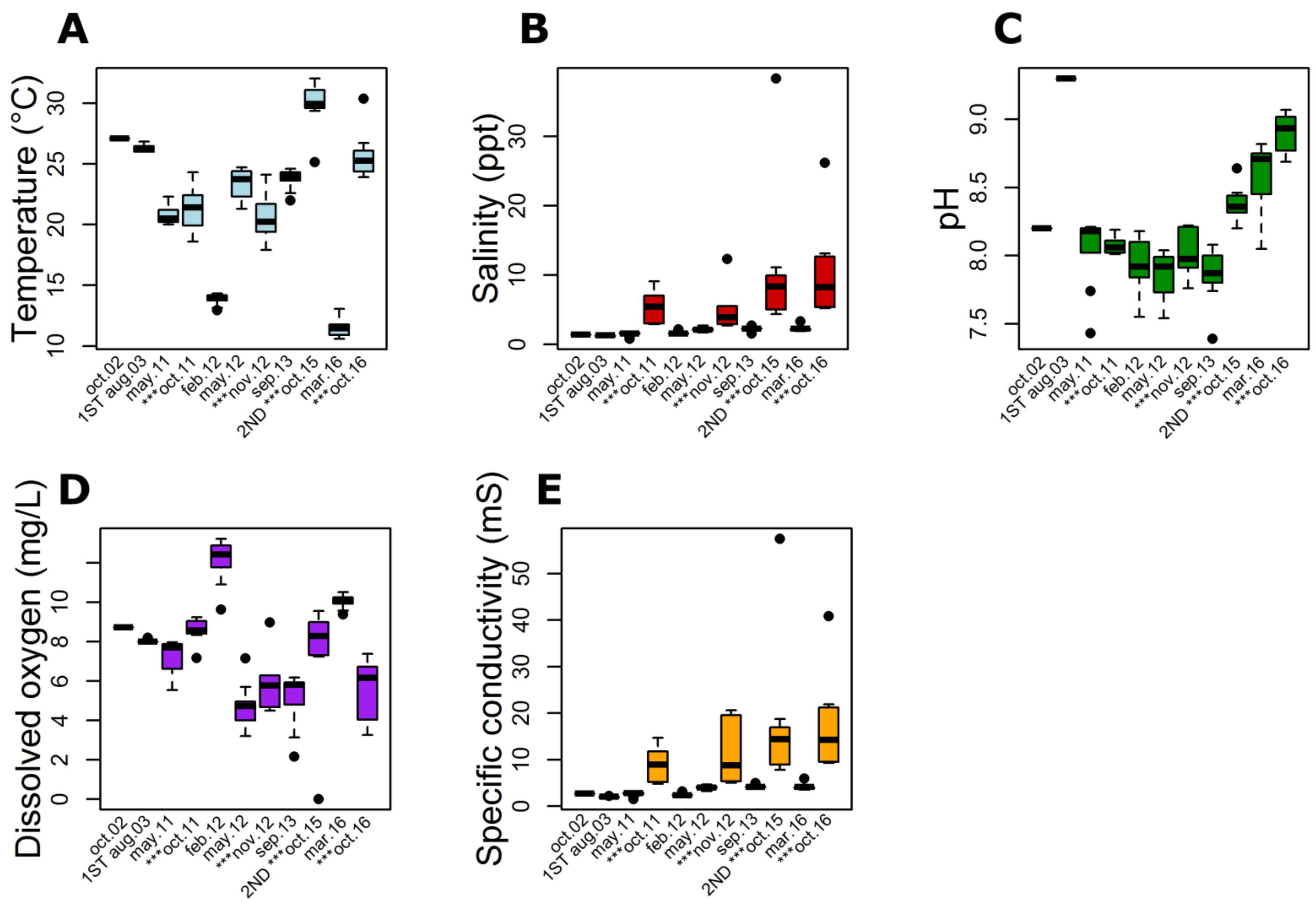

Figure S1. Barplots of environmental measurements from Laguna Intermedia since 2002 to 2016. (A) Temperature, **(B)** Salinity, **(C)** pH, **(D)** Dissolved oxygen and **(E)** Specific conductivity. Measurements before 2015 were taken by Escalante et al. (2009), Cerritos et al. (2011), Ponce-Soto et al. (2015) and García-Ulloa et al. (2019). Desiccation events are indicated by "***" on the X-axis. Samplings of P. otitidis are indicated by "1ST" and "2ND" on the X-axis.
