# Supplementary material for: Evolutionary Rescue of an Environmental Pseudomonas otitidis in Response to Anthropogenic Perturbation
Source: Front Microbiol. 2021 Jan 18;11:563885. doi: 10.3389/fmicb.2020.563885 (PMC7856823; doi:10.3389/fmicb.2020.563885)
Supplement: Supplementary file 2 [file Image_2.PDF]

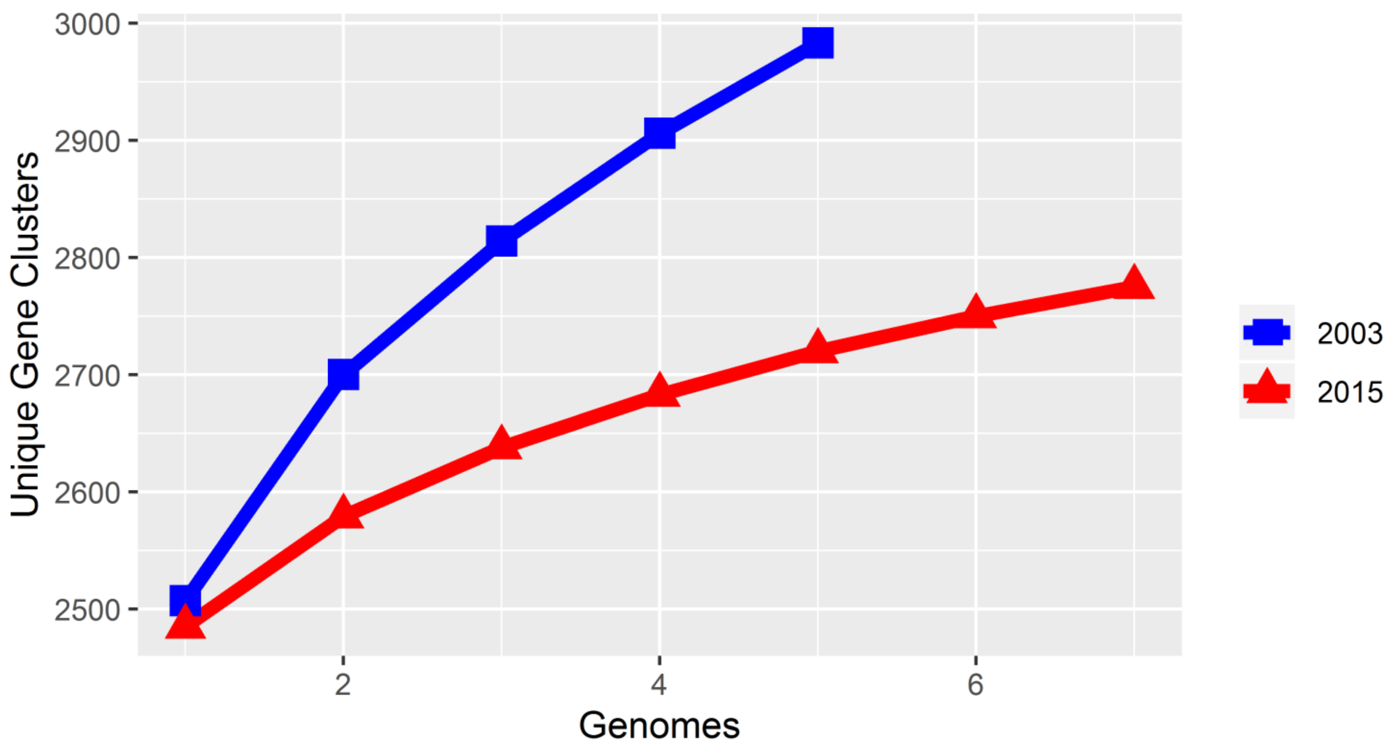


**Figure S2. Rarefaction curves of unique gene clusters from 2003 and 2015 population samples.** Blue squares and red triangles represent the 2003 and 2015 population samples, respectively. Analysis was done with the dclust algorithm from the R-package micropan v 2.0.1 (Snipen and Liland, 2015) on hmmer domains of each accessory genome. 1000 permutations were performed.
