# Supplementary material for: Evolutionary Rescue of an Environmental Pseudomonas otitidis in Response to Anthropogenic Perturbation
Source: Front Microbiol. 2021 Jan 18;11:563885. doi: 10.3389/fmicb.2020.563885 (PMC7856823; doi:10.3389/fmicb.2020.563885)
Supplement: Supplementary file 3 [file Table_1.PDF]

ID	Size (pb)	Coverage	#Contigs	N50	%GC	%BUSCOs	CDS
9M	5903084	30.8	215	68790	62.27	99.5	5361
10M	6193163	36.4	151	108181	62.2	99.5	5698
12M	5961052	30	298	63867	66.23	99.6	5402
13M	6481057	35.6	787	25766	65.64	92.4 (10 missing)	5896
15M	5978614	32.5	127	127380	66.09	99.5	5423
32M	5885926	44.4	144	89000	66.27	99.5	5286
34M	5934935	46.4	110	139000	66.35	99.5	5417
35M	5814093	48	111	119392	66.29	99.5	5226
36M	5813027	31	116	119141	66.29	99.5	5238
38M	5886822	46	104	122115	66.28	99.5	5277
39M	5878126	47	138	95388	66.27	99.5	5340
41M	5886257	32	100	118241	66.29	99.5	5271

Table S1. Assembly statistics and validation. Genomic reads were assembled with MaSuRCA 3.2.4 (Zimin et al., 2013) and SPAdes 3.10.0 *(--trusted_contigs* option) (Bankevich et al., 2012). Assembly size, N50, %GC and #Contigs were obtained with Quast v4 (Gurevich et al., 2013). Integrity of assemblies was calculated with BUSCO v2 (Simão et al., 2015) and the number of coding sequences (CDS) was obtained with Prokka 1.11 (Seemann et al., 2014).
