# Supplementary material for: Evolutionary Rescue of an Environmental Pseudomonas otitidis in Response to Anthropogenic Perturbation
Source: Front Microbiol. 2021 Jan 18;11:563885. doi: 10.3389/fmicb.2020.563885 (PMC7856823; doi:10.3389/fmicb.2020.563885)
Supplement: Supplementary file 4 [file Table_2.PDF]

	Year	Generations	Average effective population size	Upper limit	Lower limit
**KEK**	2003	4701.301	3.33E+06	9.61E+06	5.14E+05
	2011	1926.597	1.08E+03	3.57E+03	5.07E+02
	2013	1222.036	9.25E+08	1.44E+09	4.54E+08
	2015	0	4.55E+08	9.77E+08	1.01E+08
**KEC**	2003	4740.855	3.28E+06	9.10E+06	5.14E+05
	2011	1987.132	1.10E+03	3.30E+03	5.06E+02
	2013	1251.538	3.96E+08	7.00E+08	9.66E+07
	2015	0	3.96E+08	7.00E+08	9.66E+07

Table S2. Values for parameters of the two best demographic models calculated by fastsimcoal2. Number of generations, effective population size and 95% confidence intervals were estimated for *Pseudomonas otitidis* on each desiccation event (2011, 2013 and 2015), for the Contraction-Expansion-Contraction (KEK) and Contraction-Expansion-Constant (KEC) models.
